# Comparison of failure rates between full-barium and striped barium distal shunt catheters: a matched case–control study

**DOI:** 10.1007/s00381-024-06747-4

**Published:** 2025-01-18

**Authors:** Sage P. Rahm, Nicholas M. B. Laskay, Samuel G. McClugage, Joshua D. Jackson, Anastasia Arynchyna-Smith, Curtis J. Rozzelle, Brandon G. Rocque

**Affiliations:** 1 Department of Neurosurgery, Division of Pediatric Neurosurgery, University of Alabama at Birmingham, Children’s of Alabama, 1600 7th Avenue South, Lowder 400, Birmingham, AL 35233 USA; 2 Department of Neurosurgery, Texas Children’s Hospital, Houston, TX USA; 3 Department of Neurosurgery, Duke University School of Medicine, Durham, NC USA

**Keywords:** Ventriculoperitoneal shunt, Distal shunt failure, Barium catheters, Striped catheters

## Abstract

**Purpose:**

We hypothesize that distal shunt catheters fully impregnated with barium are more prone to failure compared to distal catheters with only a barium stripe. We sought to evaluate this distinction using a matched case–control study.

**Methods:**

Patient records over an 8-year period were queried for distal shunt revisions for fracture or disconnection (cases). A control group of patients with confirmed functioning distal catheters was queried from the same period and matched based on patient age at exploration/revision. Data were collected via chart review, including demographics, hydrocephalus etiology, distal catheter type, and patient age at revision. Independent *T*-test, chi-squared, and binomial logistic regression analyses were performed.

**Results:**

There were 194 patients included in the study: 97 patients with distal shunt revision and 97 controls with a functional distal shunt system. The mean patient age at distal catheter revision was 12.87 ± 4.59 years, and the mean patient age of the control group was 12.81 ± 4.59 years. The most common etiology was premature intraventricular hemorrhage (32%). Of the distal failures, 41.2% had fully impregnated barium catheters, while 58.8% had striped barium catheters. Of the control group, 76/97 (78%) patients had barium-striped distal shunt catheters and 21/97 (22%) had fully impregnated catheters. Logistic regression analysis showed that fully impregnated catheters were more likely than striped barium to fail, *p* = 0.004 (OR = 2.54, 95% CI 1.35–4.77).

**Conclusion:**

In a matched case–control format, odds of failure of fully impregnated distal catheters were 2.54 greater than striped barium catheters.

## Introduction

The placement of ventriculoperitoneal shunts (VPS) for the treatment of hydrocephalus is one of the most frequent neurosurgical procedures performed in the pediatric population. However, shunt malfunction is common, and failure occurs in most patients within 2–4 years of shunt placement [1–3]. The etiologies of shunt failure can be generally categorized by temporal (early versus late) and spatial (proximal versus distal) relationships. It should be noted that obstruction, usually proximal in nature, can occur at any time after shunt placement and remains the dominant problem with early or late shunt failure [1, 3]. In contrast, mechanical failures—namely disconnection, migration, fracture of the system—typically occur multiple years after placement [4–6].

The propensity for shunts to fracture in the neck and clavicular region reflects the repetitive dynamic mechanical forces experienced in these areas and accumulate with the age of the distal shunt catheter [4, 5, 7]. The progressive formation of micro-fissures and biochemical degradation of the superficial surface of the catheter promote precipitation of hydroxyapatite within this region, increasing rigidity and predisposing the shunt to fracture [4, 8]. Furthermore, calcification of the subcutaneous tract reduces mobility, promoting migration or disconnection from the valve [7]. There have been several anecdotal reports, including at our institution, as well as published case reports, describing a higher risk of calcification, fracture, and disconnection with catheters fully impregnated with barium [4, 9]. While barium-containing catheters allow for radiographic visualization of the distal shunt catheter, their role in promoting distal malfunction remains incompletely understood. Barium-impregnated shunt catheters are manufactured as either fully impregnated shunts, which have a homogeneous distribution of barium along the length of the catheter, or striped shunts with a thin line of barium along the length of the catheter.

As barium has been implicated in promoting calcification formation and subsequent fracture or disconnection, it has been suggested that barium-striped catheters (with a lower total content of barium) may be less prone to these types of malfunctions. However, to date, no patient-derived data have been presented to validate this claim. Hence, the purpose of this study, using a retrospective matched case–control format, is to assess the relationship between fully impregnated and barium-striped distal shunt catheters and their association with malfunction.

## Methods

### Cohort selection and data collection

A retrospective chart review was performed of the Children Hospital of Alabama (COA) hydrocephalus database for shunt surgeries between 2008 and 2016. The primary focus of the study was to compare patients with distal catheter failure to those with confirmed normal distal catheter function. The case group was identified as patients who had a shunt revision for distal catheter fracture or disconnection. All patients in this group underwent shunt revision surgery after distal catheter fracture or disconnection was diagnosed on imaging. All patients had confirmed distal catheter fracture or disconnection by direct inspection of the shunt at the time of the revision surgery, and in all cases, a new distal catheter was placed. A control group was identified of patients whose distal catheters were confirmed to be intact and functional. In order to match based on age, these patients were identified at the time of proximal shunt revision surgery to be without distal catheter failure. Specifically, these patients had imaging and direct testing of the distal catheter at the time of a proximal shunt revision operation. All of the control group had occluded proximal catheters and were treated with proximal catheter replacement. All patients at our institution with shunt failure undergoing shunt exploration have their distal shunt systems tested to make sure the distal catheter is functioning prior to completing the procedure. Thus, the proximal shunt revision patients are ultimately a way to identify patients who have not had distal failures—making them an appropriate control group for this study. The database was queried for proximal catheter revisions over the same timeframe to serve as a control group. These control patients did not have distal failure and had never undergone a distal shunt revision. Patients were matched 1:1 based on patient age at time of shunt revision. When more than one patient in the database was a match for a patient from the case group, the matched control patient was randomly selected. All patients in the case group were selected at the time of their first distal catheter event, excluding subsequent events.

Inclusion criteria included history of ventriculo-, subdural-, or cysto-peritoneal shunt placement and history of distal shunt revision for fracture or disconnect of the distal catheter (symptomatic or incidental). Exclusion criteria included distal shunt revision for reasons other than fracture or disconnection (e.g., infection) and pleural or atrial distal catheter placement. Information was collected on each patient via chart review, including general demographics, type of distal shunt catheter (fully impregnated or barium-striped), age of distal catheter (time from placement to revision), patient age at revision, patient symptoms at revision, hydrocephalus etiology (categorized by Hydrocephalus Clinical Research Network (HCRN) classification). Fractures were defined as any discontinuity in the distal shunt catheter. Disconnections were defined as a separation of the distal shunt catheter from the distal valve without any part or piece of catheter remaining on the valve. Mechanical failure of the distal shunt catheter was confirmed on imaging review of shunt series x-rays taken during patient admission for surgical revision. The exact date of shunt placement was recorded when possible. When not available, the patient’s birth date was used, assuming that for the majority of children, shunts are placed relatively close to the time of birth. The error in the age of the shunt that this approximation introduces is likely negligible, given that the index revision for this study is typically several years after initial shunt placement [10–13].

### Data analysis

Categorical variables were compared using a chi-squared analysis. Continuous variables were assessed using an independent, two-sided *T*-test. Binomial logistic regression analysis was performed on the matched case–control group to assess odds of failure between catheter types. All analyses were performed using SPSS (IBM SPSS Statistics, v24).

## Results

### Demographics

**Fig. 1:**
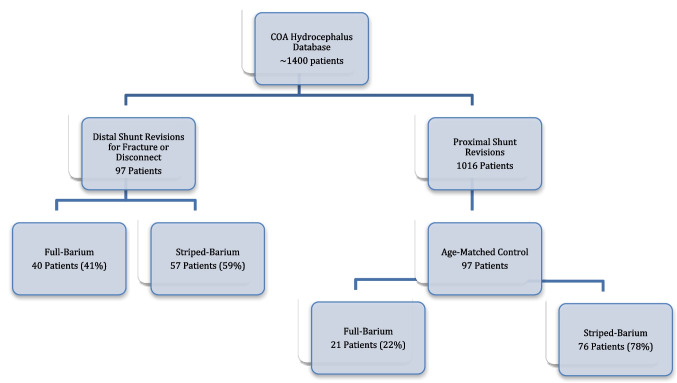
Patient selection

Over an 8-year period, 97 patients underwent a distal shunt revision for catheter fracture or disconnection and were included in the study. Over the same timeframe, 1016 patients underwent a proximal shunt revision, 97 of which were selected as an age-matched control group (Fig. 1). None of these patients had a history of distal failure. Cases and controls were matched on age at the time of shunt revision.

Sex, race, and etiology of hydrocephalus were similar between groups (Table 1). Among cases with distal shunt failures, mean distal catheter age at time of revision was 10.86 ± 5.57 years and mean patient age at revision was 12.87 ± 4.59 years. In the control group, mean patient age at proximal revision with distal catheter testing was 12.81 ± 4.59 years. No significant differences were noted between case and control groups in terms of mean patient age at revision, patient sex, patient race, or etiology of hydrocephalus.

**Table 1:** Patient allocation showing breakdown of the control group and the distal revision group (cases). Patient demographics (*n* = 194)

	Distal revision (*n* = 97)	Control (*n* = 97)	*p*-value
Patient age at shunt revision			
Mean ± SD	12.87 ± 4.59 years	12.81 ± 4.59 years	0.93
Sex, *n* (%)			0.46
Male	61 (63%)	56 (57.7%)	
Female	36 (37.1%)	41 (42.3%)	
Race			0.62
White	67 (69%)	65 (67%)	
Black	26 (26.8%)	30 (30.9%)	
Unknown	4 (4.1%)	2 (2.1%)	
Hydrocephalus etiology			0.60
Premature IVH	31 (32%)	33 (34%)	
Myelomeningocele	24 (24.7%)	25 (25.8%)	
Communicating congenital	21 (21.6%)	15 (15.5%)	
Post-infectious	5 (5.2%)	2 (2.1%)	
Aqueductal stenosis	3 (3.1%)	2 (2.1%)	
Spontaneous IVH	3 (3.1%)	6 (6.2%)	
Dandy-Walker/posterior fossa cyst	3 (3.1%)	2 (2.1%)	
Trauma	2 (2.1%)	2 (2.1%)	
Encephalocele	2 (2.1%)	0 (0%)	
Other intracranial cyst	1 (1.0%)	1 (1%)	
Other etiology	2 (2.1%)	6 (6.2%)	
Supratentorial tumor	0 (0%)	1 (1%)	
Other congenital	0 (0%)	1 (1%)	
Craniosynostosis	0 (0%)	1 (1%)	

### Comparison of fully impregnated and barium-striped catheters

Of the distal failures, 57/97 (59%) patients had barium-striped distal catheters, while 40/97 (41%) had fully impregnated distal catheters. Of the control group, 76/97 (78%) patients had barium-striped distal shunt catheters and 21/97 (22%) had fully impregnated catheters (Table 2). Logistic regression analysis showed that fully impregnated catheters had 2.5 times higher odds of fracture or disconnection compared to striped barium catheters (OR = 2.54, 95% CI 1.35–4.77, *p* = 0.004).

**Table 2:** Cross-match analysis showing the percentage of distal shunt revisions (cases) with either full-barium or barium-striped distal catheters along with the percentage of those without distal catheter failure (controls) with either full-barium or barium-striped distal catheters. Cross-match 2 × 2 table

Exposure	Cases (distal failure)	Age-matched controls (no distal failure)
Full barium	40 (41%)	21 (22%)
Barium striped	57 (59%)	76 (78%)

## Discussion

In this case–control study, we have compared a group of children experiencing distal catheter failure (case group) to a matched group of children without distal catheter failure (control group). The exposure of interest is full-barium impregnation of the distal catheter versus barium-striped distal catheter. We show that fully impregnated catheters had 2.5 times higher odds of distal failure compared to barium-striped catheters. Fully impregnated catheters represented a significantly higher proportion of the distal catheters in the distal revision group compared to the control group, suggesting a higher risk of fracture or disconnection. To date, this study represents the strongest clinical evidence that fully impregnated catheters are more prone to distal malfunction from fracture and/or disconnection than barium-striped catheters.

The concept that fully impregnated distal shunt catheters may be more prone to problems is not new. The earliest evidence noting the existence of calcification in and around catheters was in 1987 by Griebel et al. and Echizenya et al. [8, 14]. Distal shunt catheters can undergo changes over time related to inflammation and stress in and around the implanted silicone catheter, and both studies showed early evidence of this phenomenon [8, 14]. The problem and hypothesis as to why fully impregnated catheters may be more prone to problems is twofold.

First, calcified tracts can form in the tissue around the length of the distal catheter, which have been suggested as a cause for tethering and possible disconnection and/or fracture, by preventing the catheters from moving freely with neck and body movements [8]. Several studies have noted that distal catheters most commonly fracture 2–4 cm above the clavicle, which fits with this being a mechanical and stress-induced phenomenon [4, 15].

Secondly, calcifications can occur within the shunt catheter itself, making it more brittle and prone to fractures [8, 9]. Yamamoto et al. studied these differing areas of calcification extensively and found that both fully impregnated and striped barium catheters were prone to forming calcified tracts in tissues around the distal catheter, but calcification within the catheter itself was unique to fully impregnated catheters [9]. Calcified tracts around the distal catheter were also more extensive in the fully impregnated catheters, compared to those in striped catheters [9]. An in vitro study shows that fully impregnated catheters have higher astrocyte adhesion counts than barium-striped catheters do [16].

Several studies have noted a decreased tensile strength and increased rigidity in catheters with extensive calcification, further supporting the evidence that the increased calcification found in the distal catheters and tracts of full-barium catheters make them more prone to fracture [4, 8, 17]. The phenomenon seems to be more common in patients who had shunts placed at a young age and for a longer period without replacement or revision [15]. Elisavich et al. found that shunt fractures related to extensive calcification and degradation typically occurred > 7 years after implantation. This fits with our findings of an average time to failure in the distal revision group of 10.9 years. Several studies have shown a significant association between increased time of catheter placement and risk for fracture or disconnection, more in line with our own findings [4, 8, 9, 15].

### Limitations

Several important limitations in this study must be noted. As with any retrospective study, missing data is an inherent problem, given a lack of sufficient clinical documentation in the patient chart. Every effort was made to clarify the date of distal catheter placement in each patient, and a full review of the operative reports from all shunt revisions prior to the index distal revision was undertaken to ensure that the correct date of distal catheter placement was documented. Despite this, the exact date of distal catheter placement could not be identified in 30/97 patients (30.9%). Sixteen of these patients had sufficient clinical documentation to reasonably approximate the date of distal catheter insertion, such as clinic notes indicating a month and year of placement, but not an exact day. In patients with no clinical documentation of a placement date (14/30 patients), we chose to use the patient’s birth date as a reasonable correlate to initial catheter placement. Clearly, this would not be the exact date that the shunt was placed; however, we felt this would reasonably approximate the date of the initial distal catheter and shunt placement, as most shunt placements in the pediatric population occur at less than 6 months of age [1, 10, 12, 15]. Despite this, we recognize the inherent bias introduced by this decision and recommend that the results be interpreted with consideration due to this bias. Additionally, because the two groups were matched based on age and etiology, the distal catheter age of the control group was assumed to be similar to the distal catheter age of the case group. Although it is unlikely that there is a significant difference in the age of the distal catheters between these two groups due to the matching process, it is possible and remains a limitation of this study. Lastly, the authors practice largely transitioned primarily to striped barium catheter placement around 2010. Therefore, there are very few more recent cases of fully barium-impregnated catheters to study.

## Conclusion

Fully barium-impregnated distal shunt catheters have 2.54 times higher odds of distal failure compared to barium-striped catheters. Though there are important limitations, this study represents the first objective comparison of these two catheter types. While fully impregnated catheters may be easier to visualize on radiographic studies, the increased risk of distal failure compared to barium-striped catheters may outweigh that benefit. It is our recommendation that surgeons consider barium-striped distal catheters as the catheter of choice when there is not a compelling reason to do otherwise.

## Data Availability

No datasets were generated or analysed during the current study.
